# Chemopreventive and Chemotherapeutic Potential of Betulin and Betulinic Acid: Mechanistic Insights From In Vitro, In Vivo and Clinical Studies

**DOI:** 10.1002/fsn3.4639

**Published:** 2024-12-03

**Authors:** Elifsu Nemli, Beyza Saricaoglu, Celale Kirkin, Gulay Ozkan, Esra Capanoglu, Solomon Habtemariam, Javad Sharifi‐Rad, Daniela Calina

**Affiliations:** ^1^ Department of Food Engineering, Faculty of Chemical and Metallurgical Engineering Istanbul Technical University Istanbul Türkiye; ^2^ Pharmacognosy Research & Herbal Analysis Services UK, Central Avenue, Chatham‐Maritime Kent UK; ^3^ Universidad Espíritu Santo Samborondón Ecuador; ^4^ Centro de Estudios Tecnológicos y Universitarios del Golfo Veracruz Mexico; ^5^ Department of Medicine, College of Medicine Korea University Seoul Republic of Korea; ^6^ Department of Clinical Pharmacy University of Medicine and Pharmacy of Craiova Craiova Romania

**Keywords:** anticancer mechanisms, apoptosis, betulin, betulinic acid

## Abstract

Betulin is a bioactive compound found in large quantities in birch bark and has a triterpene pentacyclic structure. Through the oxidation of betulin, betulinic acid is obtained, which is found in large quantities in nature. Betulin and betulinic acid have multiple pharmacological properties such as antiviral, anti‐inflammatory, and anticancer properties. This comprehensive review aims to deepen the knowledge of the chemopreventive and chemotherapeutic effects of betulin and betulinic acid by presenting in vitro, in vivo, and clinical studies evaluating the anticancer mechanisms of betulin, betulinic acid, and their derivatives. The databases searched using specific MESh terms to conduct this review were PubMed/MEDLINE, Web of Science, TRIP database, Wiley, and Scopus. The anticancer properties of betulin and betulinic acid have been reported in a variety of experimental pharmacological studies using different types of cancer cell lines. It has been indicated that induction of apoptosis is the primary anticancer activity of these compounds by selectively affecting cancer cells. As shown by various research, the apoptotic cell death by these compounds is mainly related to factors such as type of cancer and cancer cell line, tumor size, source of betulin/betulinic acid, dose, treatment time, and the type of the drug delivery system employed. Numerous preclinical pharmacological studies and clinical trials on the chemopreventive and antitumour effects of betulin, betulinic acid, and their derivatives have been published. Future translational pharmacological studies establishing the exact anticancer dose effective in humans are needed.

## Graphical abstract

Betulin and betulinic acid exhibit anticancer properties through mechanisms including the induction of autophagy, promotion of apoptosis, and anti‐inflammatory effects. These compounds enhance autophagy, facilitating cellular degradation and recycling processes, while selectively inducing apoptosis in cancer cells. Their anti‐inflammatory actions further support their potential therapeutic applications in cancer treatment.

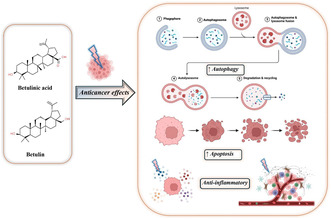

## 1 Introduction

**FIGURE 1:**
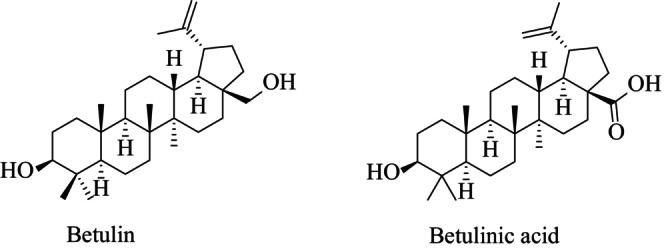
Chemical structures of betulin and betulinic acid.

Today, cancer is a critical and important public health problem that accounts to one in seven deaths in the world. The mortality rate due to cancer is higher than deaths caused by all immunodeficiency syndrome (AIDS), tuberculosis, and malaria (Cháirez‐Ramírez et al. 2016; Jain et al. 2021; Ozkan et al. 2023; Sharifi‐Rad et al. 2020). Various therapeutic approaches such as chemotherapy, radiation therapy, surgery, targeted therapy, and immunotherapy are available to treat cancer but they all have undesirable side effects (Schwiebs and Radeke 2018). Hence, the search for novel plant‐based drugs with better potency and minimal side effects has been the subject of intense research over the years. Anticancer treatment approaches using natural products used in traditional medicine have also been gaining popularity (Ali‐Seyed et al. 2016; Dhyani et al. 2022). In this respect, it has been shown that botulin (3β‐lup‐20(29)‐en‐3,28‐diol), betulinic acid (3β, hydroxy‐lup‐20(29)‐en‐28‐oic acid), and their derivatives have shown promise as anticancer agents (Csuk 2012). They both possess a pentacyclic triterpenoid structure (Figure 1) and are generally extracted from plant sources, such as birch bark ( *Betula alba* ) (Fulda 2008). The first isolation of betulin was performed in 1788 by Lowitz, while betulinic acid was first isolated in 1902 by Retzlaff (Pai and Joshi 2014; Schwiebs and Radeke 2018; Grymel, Zawojak, and Adamek 2019). They are mostly present in the *Betulaceae* family (like *Betula alba* , *B. pendula* , *B. pubescens* , *B. platyphylla* , and *B. maximowicziana* ) and can exist in the wax of leaves or peels at low amounts (less than 0.1% of a plant at dry weight) (Csuk 2012; Pai and Joshi 2014; Hordyjewska et al. 2018). The outer bark of birch tree contains high amounts of betulin between 10% and 30% (Shi, Tang, and Yan 2014), whereas the content of betulinic acid exists in the bark is lower (2% to 3%) (Jiang et al. 2021). Several other plant sources that can be used for the isolation of betulinic acid were listed by Ali‐Seyed et al. (2016). Betulin has a reported solubility in water of approximately 0.08 μg/mL, while betulinic acid is less soluble, at around 0.02 μg/mL (Shi, Tang, and Yan 2014; Wang, Soica, and Wenz 2012). The structural difference at the C‐28 position, with betulin having a hydroxyl group and betulinic acid a carboxyl group, impacts their solubility in water. While both compounds exhibit potent pharmacological properties, including anticancer, antiviral, and anti‐inflammatory effects, their poor solubility in aqueous media significantly limits their bioavailability and, consequently, their therapeutic potential. To overcome this challenge, various derivatives of betulin and betulinic acid have been synthesized to improve their solubility, enhance bioavailability, and increase their overall effectiveness as therapeutic agents. Additionally, these compounds can now be extracted from a wider variety of plants, including *Diospyros* spp., *Inga punctata*, *Sarracenia flava* , *Syzygium* spp., *Vauquelinia corymbosa* , and *Ziziphus* spp. (Ali‐Seyed et al. 2016). The anticancer potentials of betulin, betulinic acid, and their derivatives have been observed in several in vitro and in vivo studies (Csuk 2014; Hordyjewska et al. 2018). The major mechanism of anticancer action of betulin and betulinic acid is the induction of apoptosis (Fulda 2008; Fulda et al. 1997; Kasperczyk et al. 2005; Schmidt et al. 1997). Interestingly, the apoptosis induction by betulin was limited to cancer cells as it is not toxic to normal cells (Hordyjewska et al. 2018). On the other hand, the low solubility of betulin in aqueous media limits its effectiveness as an anticancer agent, but using derivatives of betulin in drug formulations can overcome this problem (Hordyjewska et al. 2018).

Even though betulin and betulinic acid have been suggested as good alternatives in cancer therapy, their anticancer mechanisms need to be understood before further drug development. Thus, this study aims to discuss their basic characteristics and anticancer mechanisms established from in vitro and in vivo studies.

## 2 Methodology

This review analyzed and summarized the anticancer molecular mechanisms, cellular targets, and signaling pathways of betulin and betulinic acid, based on preclinical experimental studies. Botanical and phytochemistry data and anticancer mechanisms were compiled by surveying online databases such as PubMed/MEDLINE, Web of Science, TRIP database, Wiley, and Scopus. For the literature search, the following MeSH terms were used: “Antineoplastic Agents/chemistry,” “Antineoplastic Agents/pharmacology,” “Apoptosis/drug effects,” “Cell Line, Tumor,” “Cell Cycle/drug effects,” “Cell Proliferation/drug effects,” “Cell Survival/drug effects,” “Dose‐Response Relationship, Drug,” “Pentacyclic Triterpenes,” “Triterpenes/chemistry,” “Triterpenes/pharmacology,” and “Triterpenes/chemical synthesis.” The most important data are summarized in Tables 1 and 2.

**TABLE 1:** Summary of in vitro studies on the anticancer potential of betulin/betulinic acid and their derivatives.

Tested compound	Tested cell lines	Outcomes	References
Betulin	Hepatoma HepG2, Hep3B cells	Prevention of cadmium‐induced apoptosis in hepatoma cells ↑Cycle arrest in G0/G1 and S phases, ↓ Cells in the G2/M phase ↓ DNA replication	Oh, Choi, and Lim (2006)
	Human lung adenocarcinoma A549 cells	↑Apoptosis ↑ Necrosis	Jae et al. (2009)
	Melanoma B164A5 cells	↓ ROS	Liu et al. (2004)
	Cervical cancer HeLa cells	↑Apoptosis, ↓Proliferation, ↑Morphological alterations ↑Cell shrinkage, ↑Karyopyknosis	Potze et al. (2014) Xu et al. (2014)
	Cervical cancer HeLa cells Hepatoma HepG2 cells Lung adenocarcinoma A549 Cells breast cancer MCF‐7 cells	↑Apoptosis ↓Cytomorphological malignant characteristics ↓Cell rounding, ↑Membrane blebbing ↓Chromatin condensation, ↑Nuclear disintegration	Li et al. (2010)
	Human SK‐BR‐3 MCF‐7 breast cancer cell	↑Apoptosis, ↓DNA synthesis ↑Permeability of the cell membranes	Orchel et al. (2021)
	Glioma cell T98G and C6 cancer cells	↓Proliferation ↓Cell viability	Król et al. (2020)
Betulin complex in γ‐cyclodextrin	Melanoma B164A5 cells	↑Cell cycle in G0/G1 and S phase	Soica et al. (2012)
	Cervical cancer HeLa cells	↑Apoptosis ↑Cycle arrest in G0/G1	Joseph et al. (2019)
	Human colorectal cancer tumor cells	↓Migration and invasion of cancer cells ↑ROS, ↑Apoptosis ↑Permeability of the cell membranes	Wang et al. (2020); Zeng et al. (2019)
	Melanoma cell lines	↓Cell viability, ↑Apoptosis	Wróblewska‐Łuczka et al. (2022)
Betulinic acid	Equine melanoma cells	↓Proliferation, ↓Cell viability	Weber et al. (2020)
	Ovarian cancer cell lines	↑Apoptosis, ↓Proliferation, Morphological cancer cells alterations No effect on normal cells	Serain et al. (2021); Wang, Liu, and Dou (2015)

*Note:* Symbols: ↑ increased and ↓decreased.

**TABLE 2:** Summary of in vivo studies on the anticancer potential of betulin/betulinic acid and their derivatives.

Tested compound	Cancer model	Xenograft animal models	Outcomes	References
Betulinic acid	Breast cancer	BALB/c athymic nude mice bearing MCF‐7 breast adenocarcinoma	↓Tumor size Dose = 50–100 mg ↓Proliferation, ↓invasion, ↓Angiogenesis No signs of toxicity	Damle, Pawar, and Narkar (2013)
	*Prostatic adenocarcinoma*	Nude mice bearing LNCaP cell xenografts	↓Tumor growth ↓Sp1, ↓Sp3, ↓Sp4, ↓VEGF ↑Apoptosis	Chintharlapalli et al. (2007)
Betulinic acid complexed with octakis‐[6‐deoxy‐6‐(2‐sulfanyl ethane sulfonic acid)]‐γ‐CD (GCDG)	Skin cancer Melanoma	C57BL/6J mice bearing murine melanoma developed by inoculation of B164A5 cells	↓Tumor volume ↓Tumor weight ↓Melanin, ↓erythema levels in mice treated with betulinic acid: GCDG 1:1 complex than the control group ↑Survival time	Soica et al. (2014)
Betulinic acid‐containing liposomes	Lung cancer and colon cancer	Athymic nude Foxn1 mice grafted with a human lung cancer cell line (A549) or human colon cancer cell line (SW480)	↓Tumor growth by oral administration of liposomes with betulinic acid ↑Survival No signs of systemic toxicity	Mullauer et al. (2011)
TPP‐linked derivative of betulin (3β‐Hydroxylup‐20 (29)‐en‐28‐yl 5‐(Triphenylphosphonio)‐pentanoate Bromide)	Chronic myeloid leukemia	Wild‐type zebrafish grafted with labeled K562 cells	↓Proliferation ↓Migration ↑Cytotoxicity	Ye et al. (2017)
Betulin complexed with octakis‐[6‐deoxy‐6‐(2‐sulfanyl ethane sulfonate)]‐γ‐CD (GCDG)	Skin cancer Melanoma	C57BL/6J mice bearing murine melanoma developed by injection of B164A5 cells	↓Tumor volume, ↓Tumor growth ↓Tumor weight ↓S100, ↓VEGF No sign of the effects of invasive cells on neighboring organs	Soica et al. (2012)
Poly(lactic‐co‐glycolic acid) (PLGA)‐loaded nanoparticles of betulinic acid	Hepatic cancer	Wistar albino rats bearing hepatocellular carcinoma	↑Plasma concentration, ↑Time for reaching the maximum plasma concentration Higher restoration in body weight, oxidative stress parameters, liver marker enzymes, and histological architecture	Kumar et al. (2018)
Betulinic acid in 6% dimethyl sulfoxide in corn oil	Colorectal cancer	BALB/c nude mice inoculated with HCT116 cells	↓Tumor volume, ↓ Tumor weight ↓Tumor growth, ↓MMP‐2+, ↓Ki‐67+ cells survival, ↑caspase‐3	Zeng et al. (2019)
Betulin containing nanoemulsion (oil‐in‐water nanoemulsion)	Skin cancer Melanoma	BALB/c mice bearing skin carcinoma	↓Skin redness ↑Active respiration of isolated liver mitochondria with the application of betulin nanoemulsion for 12 weeks together with DMBA (7,12‐dimethylbenz[a]anthracene) and TPA (12‐O‐tetradecanoylphorbol 13‐acetate) ↑Transepidermal water loss, ↓skin damage, ↓skin tumor apparition ↓Inflammatory process	Dehelean et al. (2013)
The polyethylene glycol‐modified (PEGylated) betulinic acid liposomes	Cervical cancer	Kunming mice bearing cervical cancer by injection of U14 cells	↓Tumor growth, ↓Tumor size, ↓Tumor weight No visible weight loss No toxicity or side effects of the nanocarriers	Liu et al. (2016)
Betulinic acid derivative NVX‐ 207	Skin cancer	Two adult female gray horses bearing at least one melanoma	No local signs of irritation or inflammation no clinical signs of systemic illness Slight deviations in hematologic and blood‐biochemical evaluations Good toleration of betulinic acid derivative NVX‐ 207	Liebscher et al. (2016)
Betulinic acid in 5% dimethyl sulfoxide in corn oil	Breast cancer	BALB/c mice bearing breast cancer by inoculated with 4 T1 cell	↓Tumor growth, ↓ Tumor weight, ↓ Proliferation ↓MMP‐2, ↓MMP‐9, ↓P‐Stat3, ↑caspase‐3 ↓Micrometastatic nodules No toxic signs	Zeng et al. (2018)
Polar glycosylated derivative of botulin (28‐*O*‐α‐L‐rhamnopyranosylbetulin 3β‐*O*‐α‐L‐rhamnopyranoside (Bi‐L‐RhamBet))	Lung cancer	C57BL/6NCrl mice bearing lung cancer inoculated with LLC1 cells	No toxicity signs ↓Tumor growth	Mihoub et al. (2018)
Encapsulated betulinic acid derivative in a polymeric nanocarrier system (2c‐NP)	Colorectal cancer	Sprague–Dawley rats Swiss albino mice bearing colorectal cancer	↑Preferential tumor selectivity of 2c‐NP Time‐dependent accumulation of 2c‐NP in the target organ (the colon) ↓Propagation of cancerous regions ↑Epithelial layer, ↑the disappearance of ulcerative regions of animals bearing colorectal cancer treated 2c‐NP No alteration in the histopathological integrity of normal colon cells	Dutta et al. (2019)

*Note:* Symbols: ↑ increased and ↓decreased.

## 3 Bioavailability and Strategies for Improving the Bioactivity of Betulin and Betulinic Acid

The chemical structures of betulin and betulinic acid share similar pharmacological properties, but their low solubility in aqueous media remains a significant limitation. Betulin has a solubility of approximately 0.08 μg/mL, whereas betulinic acid is even less soluble, around 0.02 μg/mL (Shi, Tang, and Yan 2014; Wang, Soica, and Wenz 2012). Despite the presence of an acidic group, the carboxyl group at the C‐28 position in betulinic acid actually decreases its solubility compared to betulin, due to increased hydrophobicity (Shi, Tang, and Yan 2014; Grymel, Zawojak, and Adamek 2019). This reduced solubility limits the bioavailability and effectiveness of betulinic acid, which has prompted the synthesis of derivatives and the development of advanced drug delivery systems to overcome this challenge. In general, the three most structural groups of these compounds which were subject to chemical modifications are the secondary hydroxy group at the C‐3 position, the primary hydroxy group for betulin or carboxylic acid group for betulinic acid at position C‐28, and the isopropenyl side chain of the triterpene backbone (Grymel, Zawojak, and Adamek 2019). The bioavailability and anticancer efficacy of betulin, betulinic acid, and their derivatives can be also enhanced by utilizing drug delivery systems with nanotechnology. For instance, nanoencapsulation and emulsification in lipophilic media can improve bioavailability (Mierina, Vilskersts, and Turks 2018). Moreover, it was reported that the mammalian target of rapamycin (mTOR) activation is important for the betulin‐induced cytotoxicity in renal cell carcinoma, as mTOR increased pyruvate kinase muscle isozyme M2PKM2 and hexokinase 2 (HK2) expression by upregulating the aerobic glycolysis (Cheng et al. 2017). On the other hand, Huyke et al. (2009) reported that a betulin‐based oleogel reduced the lesions when used as a topical treatment of actinic keratoses in a clinical study. Overall, it is observed that the chemical modifications and/or nanoformulations of betulin and betulinic acid are good ways for improving their limitations like low solubility and bioaccessibility. However, more in vitro and in vivo studies should be performed for understanding and further improvements by such modifications.

## 4 Anticancer Effects of Betulin and Betulinic Acid

### 4.1 Molecular Mechanisms of Action

**FIGURE 2:**
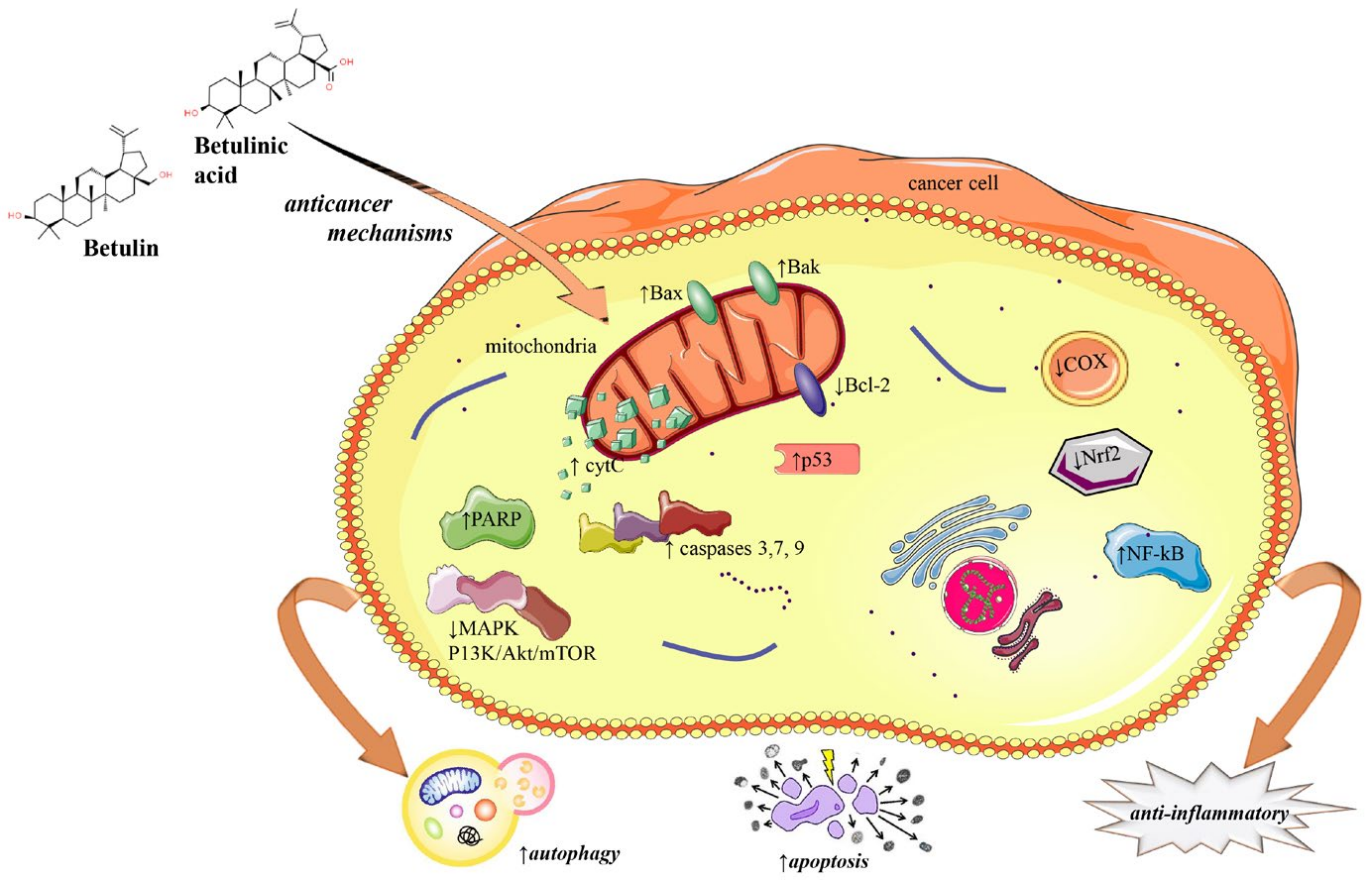
Potential anticancer and anti‐inflammatory molecular mechanisms of betulin and betulinic acid. Symbols: ↑upregulated and ↓downregulated. Abbreviations: Apoptosis regulator (Bax), poly(ADP‐ribose) polymerase (PARP), mitogen‐activated protein kinases (MAPKs), cyclo‐oxygenase‐2 (COX‐2), transcription factor nuclear factor erythroid 2–related factor 2 (NRF2), and nuclear factor kappa‐light‐chain‐enhancer of activated B cells (NF‐κB). The figure on the left indicates anticancer activity via induction of autophagy and apoptoses, while on the right side depicts anti‐inflammatory effect by suppressing proinflammatory mediators (NF‐κB and COX) while boosting NRF2 (antioxidant/anti‐inflammatory).

The basic anticancer mechanism of many anticancer agents is generally explained by induction of apoptosis (Elmore 2007; Fulda 2008; Fulda and Debatin 2006; Król et al. 2015; Plati, Bucur, and Khosravi‐Far 2008). Apoptosis has two pathways: extrinsic (led by death receptors) and intrinsic (led by the mitochondria) (Fulda 2008; Fulda and Debatin 2006). Thus, one or both of these pathways may be activated by anticancer agents. The extrinsic pathway is related to death receptors, such as CD95, tumor necrosis factor receptor 1 (TNFR1), TNF‐related apoptosis‐including ligand‐receptor 1 (TRAIL‐R1), and TRAIL‐R2 (Fulda and Debatin 2006). The intrinsic pathway includes the permeabilization of the outer mitochondrial membrane and the release of proteins, such as cytochrome C, second mitochondria‐derived activator of caspase (Smac/DIABLO), Omi/HtrA2, AIF, and endonuclease G, which leads to the activation of caspases (Fulda and Debatin 2006). It was reported that betulin and betulinic acid induce anti‐inflammatory and anticancer activity by inhibiting the nuclear factor‐κB (NF‐κB)–mediated inflammatory mechanisms, inhibiting the conversion of arachidonic acid to prostaglandins by cyclooxygenase enzymes, and modulation of the nuclear factor erythroid 2–related factor 2 (Nrf2) phosphorylation and other antioxidant defense system (Tuli et al. 2021) (Figure 2).

#### 4.1.1 Anticancer Mechanism of Betulin

Betulin causes apoptotic cell death by changing the mitochondrial membrane potential and releasing mitochondrial cytochrome c (Li et al. 2010). The induction of apoptosis triggered by betulin was associated with the activation of caspase‐9, ‐3/‐7, mitochondrial translocation of Bax and Bak, and cleavage of poly (ADP‐ribose) polymerase (PARP) (Li et al. 2010). In addition to activating the caspase pathways, betulin can decrease the phosphorylation of mitogen‐activated protein kinase (MAPKs) and cause autophagy via AMP‐activated protein kinase AMPK and phosphatidylinositol‐3‐kinase (PI3K)/AKT/mTOR signaling (Bębenek et al. 2020).

#### 4.1.2 Anticancer Mechanism of Betulinic Acid

It was stated that betulinic acid demonstrates melanoma‐specific cytotoxicity (Pisha et al. 1995). It has also been observed that betulinic acid could be effective against several types of cancer (Cichewicz and Kouzi 2004). The possible mechanism of anticancer action of betulinic acid can be explained by the induction of apoptosis or upregulation of p53 in metastatic melanoma cells (Cichewicz and Kouzi 2004; Schmidt et al. 1997). The apoptosis induced by betulinic acid is not affected by the status of the p53 gene (Fulda et al. 1997). Apoptosis is induced by betulinic acid due to the changes, such as the loss of the mitochondrial membrane potential, modulation of expression of Bcl‐2 proteins, and activation of NF‐κB (Fulda 2008; Schmidt et al. 1997). The anticancer effect of betulinic acid on colorectal cancer was evidenced by the inhibition of glycolytic and glutaminolysis enzymes, as these pathways favor the growth of tumor cells (Wang et al. 2019). Moreover, it was reported that betulinic acid and betulin displayed different anticancer mechanisms against non‐small cell lung cancer cell lines (A549, H358, NCI–H1703) under hypoxic conditions, and betulinic acid accelerated apoptosis and decreased the clonogenic potential of the cancer cells (Kutkowska, Strzadala, and Rapak 2021).

## 5 Preclinical Pharmacological Studies on Anticancer Properties of Betulin and Betulinic Acid

### 5.1 In Vitro Studies

The summary of many studies conducted to show the anticancer properties of betulin and betulinic acid in different cancer cells is shown in Table 1. In one study using the human hepatoma cell lines, HepG2 and Hep3B cells, betulin was employed to examine how it modulates the cadmium‐induced apoptosis (Oh, Choi, and Lim 2006). Treatment of HepG2 cells resulted in a reduction in the number of cells in the G2/M phase as well as cell cycle arrest in the late G0/G1 and early S phases. On the other hand, betulin only suppressed the G2/M phase in Hep3B cells. Hence, the effect of betulin may be dependent on cancer cell types. Additionally, the study hypothesized that betulin would cause a brief decrease in DNA replication without affecting the genes p21 and p53 that control cell cycle. Another study evaluated the anticancer properties of betulin complex with γ‐cyclodextrin and observed that the betulin complex stops the cell cycle of murine melanoma B164A5 cells in the S phase at a concentration of 10 M. At the same time, G0/G1 phase had fewer number of cells (Soica et al. 2012).

Betulin treatment also induces apoptosis in the A549 cell line (human lung adenocarcinoma cells), according to the study by Jae et al. (2009). After the treatment of cells with the compound, nearly equal numbers of necrotic and apoptotic cells were discovered. By rapidly increasing the generation of reactive oxidative species and concurrently dissipating mitochondrial membrane potential in a dose‐ and time‐dependent manner, betulinic acid also has an anticancer effect on melanoma cells (Liu et al. 2004). According to another study, after 24‐h exposure to betulin and depending on the dose, the suppression of HeLa cells' ability to proliferate was also accompanied by typical morphological alterations such as cell shrinkage and karyopyknosis (Potze et al. 2014). Similar studies also found that betulinic acid exhibits anticancer properties in HeLa cells. Betulinic acid exerted this effect by increasing cell apoptosis, suppressing proliferation, and causing morphological alterations in the mitochondrial membrane (Xu et al. 2014). According to a study, betulin causes inhibition of cell viability of cervix carcinoma HeLa cells, hepatoma HepG2 cells, lung adenocarcinoma A549 cells, and breast cancer MCF‐7 cells (Li et al. 2010). When the anticancer mechanism of botulin was investigated, it was obtained that cytomorphological characteristics that change with betulin treatment are also indicative of apoptotic cells. Cell rounding, membrane blebbing, chromatin condensation, nuclear disintegration, and the creation of apoptotic structures are some examples of this. This study further establishes that, in contrast to betulinic acid, betulin did not directly cause the release of mitochondrial cytochrome C in isolated mitochondria. Numerous investigations have shown that betulinic acid selectively suppresses the proliferation of ovarian cancer cell lines while not affecting normal cells (Serain et al. 2021; Wang, Liu, and Dou 2015). The anticancer activity of betulinic acid on ovarian cancer cells is time‐ and dose‐dependent and based on an increase in cell apoptosis, a reduction in cell proliferation, and morphological alterations to the mitochondrial membrane (Liao et al. 2020; Serain et al. 2021; Wang, Liu, and Dou 2015). In addition to this, betulinic acid shows a synergistic effect when it is treated with 5‐fluorouracil on ovarian cancer cells (Wang, Liu, and Dou 2015).

### 5.2 In Vivo Studies

The anticancer potentials of betulin and betulinic acid as well as their mechanisms of action against different cancer types were also established through in vivo studies (Table 2).

The first study about the antitumor activity of betulinic acid in vitro and in vivo was performed by Pisha et al. (1995). The results of this study and others confirmed that betulinic acid treatment suspended the growth of melanoma both in vitro and in mice (Hordyjewska et al. 2018, 2019). In the study of Damle, Pawar, and Narkar (2013), the administration of betulinic acid resulted in a significant reduction in the tumor size, and in cell proliferation, invasion, and angiogenesis; also delaying tumor formation in nude mice bearing breast cancer. Moreover, any toxicity or body weight loss has not been observed in mice treated with betulinic acid in comparison with control animals. Similarly, inhibition in tumor growth of athymic nude mice bearing LNCaP cell xenografts has been observed by betulinic acid treatment and this result was associated with reduction in the expression of Sp1, Sp3, and Sp4 proteins and vascular endothelial growth factor (VEGF) and increased apoptosis in tumors (Chintharlapalli et al. 2007). Another study showed that treatment of the TPP‐linked derivative of betulin exhibited inhibitory effect on the proliferation and migration of K562 cells of zebrafish and provided higher in vivo activity in comparison with that of betulin (Ye et al. 2017).

## 6 Clinical Studies Related to the Anticancer Activity of Betulin and Betulinic Acid

Plant‐derived compounds have proven their promise as anticancer or chemopreventive agents (Król et al. 2015). The anticancer and cytotoxic activity of betulin and betulinic acid have been investigated through various in vitro and in vivo studies, and they have demonstrated great potential for treating several human cancer types. They have great potential for the treatment of several human cancer types. The significative and characteristic qualities and activity of betulinic acid further attracted the attention to undertake clinical trials. The compound also aroused the interest of the National Cancer Institute's (NCI) Rapid Access to Intervention Development (RAID) program (Ali‐Seyed et al. 2016). A phase I/II clinical trial between 2006 and 2013 was performed with 28 patients for the evaluation of the safety and effectiveness of ointment containing 20% betulinic acid (BA ointment) as a treatment for dysplastic nevi which has a transformation potential into melanoma (NCT00346502, Clinical Trials database). In this phase I/II clinical trial, daily application of 20% BA ointment for 4 weeks was planned to be followed by surgical removal of the tumor for comparison with untreated dysplastic nevi. However, this clinical trial was suspended in 2013 due to funding issues and its results were not published. Another phase 1 pilot clinical trial was carried out with 12 participants with cutaneous metastatic melanoma for the evaluation of the safety, tolerability, and effectiveness of ALS‐357 (betulinic acid—BA) according to the NCT00701987, Clinical Trials database (Chemspider 2022; WFO 2021). However, the study did not provide any information about the concentration of betulinic acid in the preparation. During this phase 1 pilot clinical trial, the drug was applied topically in ointment form for 4 weeks in four groups (twice a week, every other day, once a day, and twice a day), and the TUNEL method was used to evaluate apoptosis in biopsies. Unfortunately, no information about the outcomes of this research has been published (Drąg‐Zalesińska and Borska 2019). According to Huyke et al. (2009), actinic keratoses (AK) are a type of squamous cell carcinoma that needs to be treated. A new topical treatment option for AK is available in the form of an oleogel made from a standardized triterpene dry extract derived from birch bark. This oleogel contains betulin and the phase 2a study was conducted at a single center and involved 45 patients who had 10 or fewer actinic keratoses (AK). The patients were randomly assigned to one of three treatment groups: topical betulin‐based oleogel applied twice daily, cryotherapy with liquid nitrogen, or a combination of cryotherapy with topical betulin‐based oleogel. After 3 months, the patients' responses to treatment were evaluated based on clinical examination. The response was classified into three categories: complete clearing of the lesions (100%), responders to therapy (≥ 75% clearing of the lesions), and nonresponders (< 75% clearing of the lesions). The use of betulin‐based oleogel as a treatment was well tolerated by patients. After 3 months of treatment, the rates of complete clearing of the lesions and > 75% clearing were as follows: 64% (86%) for betulin‐based oleogel (*n* = 14), 79% (93%) for cryotherapy (*n* = 14), and 71% (71%) for the combination therapy (*n* = 14). Biopsies taken before and after treatment from 8 patients in the study showed a reduction in the degree of dysplasia in the epidermis in all treatment groups. The use of betulin‐based oleogel appears to be a promising and effective new method for treating actinic keratoses topically (Huyke et al. 2009). Moreover, for the evaluation of the effectiveness of Oleogel‐S10 in the treatment of actinic keratosis and planning of phase III trials, a multicenter, randomized, double‐blind phase II trial was performed between 2008 and 2010 (NCT00786994, Clinical Trials database) (Chemspider 2022; WFO 2021). According to the results of this study, including two previous clinical trials, Oleogel‐S‐10 demonstrated effectiveness and was well tolerated. In a subsequent phase II trial, which was open‐label, Oleogel‐S‐10 alone was found to be similarly effective as a more invasive treatment involving cryotherapy. The study reported response rates of 85%, with a clearance of over 75% of lesions in patients treated with Oleogel‐S‐10 over 3 months. These promising results prompted a new phase II trial, which is randomized, multicenter, and double‐blind, with a placebo control group using petroleum jelly. This new trial is designed to produce more reliable results to plan a phase III study. However, according to the result of a clinical trial performed by Pflugfelder et al. (2015) with 165 patients treated topically for 3 months in a four parallel study as Oleogel‐S10 once daily, Oleogel‐S10 twice daily, or placebo (petroleum jelly) once or twice daily, the treatment with Oleogel‐S10 did not show better efficacy for the treatment of actinic keratosis than the placebo group although the drug was well tolerated. In addition to all these studies, in the study of Willmann et al. (2009), NVX‐207, having advantageous chemical and pharmacological properties, is a novel semi‐synthetic betulinic acid‐derived drug. A phase I/II clinical study was performed in canine cancer patients suffering from spontaneously arising pretreated tumors to evaluate the tolerability and efficacy of NVX‐207. After the local treatment of NVX‐207 (10 mg mL^−1^), clinically excellent responses were obtained in all five dogs that suffered from naturally occurring cancer. There were no systemic side effects but minor local side effects were found after the tumor and surrounding tissue was infiltrated. Unfortunately, there are not many clinical trials with betulin, betulinic acid and their derivatives, especially for the direct treatment of cancers in human patients. Consequently, more clinical trials performed with human patients for different cancer types and assessment of information resulting from the clinical trials are needed.

## 7 Therapeutic Perspectives, Clinical Gaps and Limitations

The anticancer activity of betulin can vary depending on some factors, such as the dose and the type of cell line and cancer (Bębenek et al. 2020; Kıran et al. 2020). Even though betulin and betulinic acid can demonstrate anticancer activity as assessed by in vitro studies, their low solubility in water can compromise their potential in cancer therapy (Drag‐Zalesinska et al. 2009).

Thus, they can be structurally modified to improve their anticancer action (Drag‐Zalesinska et al. 2009). It was also stated that betulin ester derivatives could exhibit a higher anticancer activity compared to betulin and betulinic acid due to their enhanced bioactivity and concentration in cancer cells (Drag‐Zalesińska et al. 2017). Similarly, the addition of sulfonamides onto betulin was found to be effective in the regulation of carbonic anhydrases and the inhibition of clonogenic survival and radiosensitization. Thus, they exhibited cytotoxic, antiproliferative, and apoptotic effects (Güttler et al. 2021). Furthermore, Wang et al. (2022) evaluated the anticancer potential of several new betulin derivatives with aromatic hydrazone side chain on the C‐28 position and noted that betulin‐28‐(indole‐4‐ylmethylene) hydrazine exhibited high antiproliferative activity against MCF‐7 breast cancer cell lines and could induce their apoptosis. It was claimed that betulinic acid could be a good alternative anticancer drug, as its anticancer activity targets the mitochondria and is independent of p53 (Ali‐Seyed et al. 2016). Also, anticancer drugs that work independently of p53 and directly on the mitochondria are less prone to drug resistance (Ali‐Seyed et al. 2016). Betulinic acid also has promising activity when combined with radiation therapy. This effect of betulinic acid and its derivatives was analyzed on glioblastoma cell lines (U251MG, U343MG, and LN229). Betulinic acid derivatives were discovered to decrease cell migration, PARP cleavage, and levels of Survivin protein expression. Additionally, after being exposed to both betulinic acid derivatives, U251MG cells showed a slight increase in their radiation sensitivity (Bache et al. 2014). It can also be a promising agent when combined with 5‐fluorouracil in colon cancer cell lines (Jung et al. 2007). Even though there have been several in vitro and in vivo studies reporting that betulin and betulinic acid exhibit good potential to be used as anticancer drugs, crude extracts of plants that are rich in these compounds, such as birch bark, could also offer a natural or traditional alternative in cancer therapy. For instance, extracts of *Hibiscus syriacus* as a source of betulin were found effective in inhibiting the viability of breast cancer cells and inducing apoptosis (Hsu et al. 2015). The number of clinical studies employing betulin and betulinic acid as anticancer agents is limited (Drąg‐Zalesińska and Borska 2019), but they have been utilized frequently in the therapy of cancers as part of traditional medicine (So et al. 2018). For instance, a mushroom (*Inonotus obliquus*) that lives in birch trees and is rich in betulinic acid content was used in traditional medicine for cancer treatment (Ali‐Seyed et al. 2016). In the analyzed literature, a higher number of studies were performed on betulinic acid compared to betulin because the solubility of betulin in aqueous media is even more limited (Hordyjewska et al. 2018). Furthermore, when the number of studies is considered, fewer in vivo than in vitro studies were performed. The reason for this has generally arisen from the low solubility and bioaccessibility of betulin and betulinic acid even if betulinic acid is more soluble than betulin. To overcome these limitations, nano‐drug delivery systems like liposomes, polymersomes, polymeric nanoparticles, and dendrimers can be preferred since the use of these systems enhance their stability, solubility, and efficacy (Liu et al. 2016). Therefore, the performed studies especially in vivo studies have been focusing in recent years on the use of betulin and betulinic acid as anticancer agents with nano‐drug delivery systems.

## 8 Conclusion

In the literature, there are diverse in vitro and in vivo studies performed to highlight the mechanisms of the action of betulin and betulinic acid against different types of cancer. Considering the research conducted with those bioactive compounds, it has been reported that the type of cancer and cancer cell line, tumor size, source of betulin/betulinic acid, dose, treatment time, and the type of drug delivery system have a potent influence on their anticancer potential. On the other hand, it should be taken into consideration that the bioactivity of those compounds mainly depends on their water solubility, bioaccessibility, and bioavailability. To improve the effectiveness of betulin owing to its low solubility, effective drug delivery systems regarding the type of carrier, carrier‐to‐betulin ratio, and other related parameters must be developed. Overall, in vitro and in vivo studies showed that there was a great potential to utilize betulin, betulinic acid, and their derivatives against different types of cancer. However, it is important to carry out more in vivo and clinical studies before using them in the treatment of cancer in humans.

## Author Contributions

**Elifsu Nemli:** data curation (equal), investigation (equal), writing – original draft (equal), writing – review and editing (equal). **Beyza Saricaoglu:** data curation (equal), investigation (equal), writing – original draft (equal), writing – review and editing (equal). **Celale Kirkin:** data curation (equal), writing – original draft (equal), writing – review and editing (equal). **Gulay Ozkan:** data curation (equal), investigation (equal), writing – original draft (equal), writing – review and editing (equal). **Esra Capanoglu:** investigation (equal), validation (equal), writing – original draft (equal), writing – review and editing (equal). **Solomon Habtemariam:** investigation (equal), supervision (equal), validation (equal), writing – original draft (equal), writing – review and editing (equal). **Javad Sharifi‐Rad:** conceptualization (equal), data curation (equal), investigation (equal), methodology (equal), project administration (equal), supervision (equal), validation (equal), visualization (equal), writing – original draft (equal), writing – review and editing (equal). **Daniela Calina:** conceptualization (equal), data curation (equal), investigation (equal), methodology (equal), project administration (equal), validation (equal), writing – original draft (equal), writing – review and editing (equal).

## Conflicts of Interest

The authors declare no conflicts of interest.

## Data Availability

Not applicable.
